# Slide tracheoplasty for repair of complex tracheoesophageal fistulas in children: A salvage technique

**DOI:** 10.1016/j.xjtc.2025.09.032

**Published:** 2025-10-13

**Authors:** Jiaxi Huang, Li Jiang, Gong Chen, Chen Chao, Weicheng Chen, Ming Ye, Shunmin Wang, Gang Chen

**Affiliations:** a Cardiovascular Center, Children's Hospital of Fudan University, Shanghai, China; b Department of Anesthesiology, Children's Hospital of Fudan University, Shanghai, China; c Department of Pediatric Surgery, Children's Hospital of Fudan University, Shanghai, China; d Department of Otolaryngology, Children's Hospital of Fudan University, Shanghai, China; e Department of Cardiothoracic Surgery, Shanghai Children's Medical Center, Shanghai Jiaotong University School of Medicine, Shanghai, China

**Keywords:** slide tracheoplasty, tracheoesophageal fistula, children, cardiopulmonary bypass

## Abstract

**Objective:**

Complex tracheoesophageal fistula (TEF), encompassing recurrent, large-defect, or foreign-body-induced TEF caused by congenital or acquired origin, represents a formidable surgical challenge. Although various endoscopic and surgical techniques have been developed, the optimal approach remains controversial. Slide tracheoplasty with cardiopulmonary bypass provides excellent exposure of both the trachea and esophagus, making it a potentially valuable salvage technique for the treatment of complex TEF in children. We present a series of patients who underwent successful slide tracheoplasty by a multidisciplinary team in our institution.

**Methods:**

We retrospectively reviewed 3 consecutive patients who underwent slide tracheoplasty for complex TEF between January and April 2024. Data collected included demographic characteristics, etiology, surgical details, perioperative parameters, and outcomes.

**Results:**

All 3 patients successfully underwent slide tracheoplasty and esophageal repair with cardiopulmonary bypass support. All patients were successfully weaned from mechanical ventilation and had an uneventful recovery. At a median follow-up of 12 months, there were no cases of postoperative infection, vocal cord paralysis, esophageal or tracheal strictures, recurrent fistula, or need for reintervention.

**Conclusions:**

Slide tracheoplasty with cardiopulmonary bypass demonstrates excellent early outcomes for the management of complex tracheoesophageal fistula in children. This technique represents a promising salvage option when conventional repair approaches are inadequate or have failed.

**Figure:**
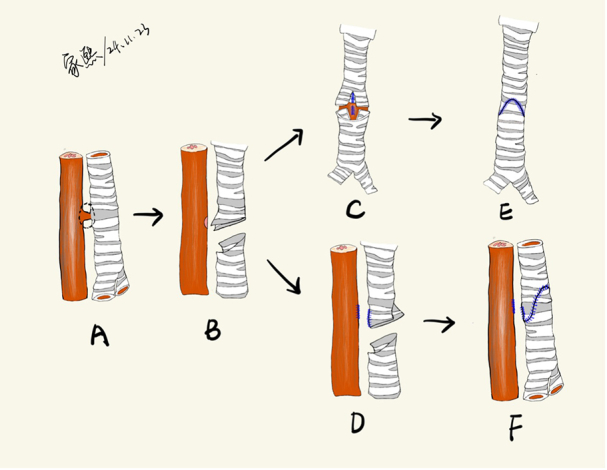
The lesion of TEF was excised and slide tracheoplasty was performed.

**Central Message**

The early efficacy of the slide tracheoplasty in the treatment of complex tracheoesophageal fistula is promising.

**Perspective**

This study aims to address the surgical challenge of TEF through an innovative protocol combining 3 key elements: CPB-assisted dual-field exposure enabling precise dissection of intricate fistulae and repair of both the trachea and the esophagus; sliding tracheal flap technique to reduce risk of postoperative tracheal stenosis; and a perioperative anti-infection protocol to avoid recurrence.

Tracheoesophageal fistula (TEF) represents a congenital or acquired pathologic condition characterized by an abnormal communication between the posterior tracheal wall and the anterior esophageal wall. Congenital TEF occurs in approximately 1 in 3000 live births, with or without associated esophageal atresia.^1^ With advances in surgical technique, congenital TEF is now routinely repaired via a thoracoscopic approach, achieving successful fistula closure rate ranging from 87% to 100%.^2^, ^3^, ^4^, ^5^, ^6^, ^7^ However, recurrent TEF, reported in 3% to 20% of infants after initial repair, presents substantial surgical challenges as the result of scarring and inflammation from previous operations.

Acquired TEF may result from foreign body ingestion such as button batteries, malignancy, iatrogenic injuries from tracheostomy or intubation, or erosion caused by tracheal or esophageal stents.^8^, ^9^, ^10^, ^11^, ^12^, ^13^, ^14^ These acquired TEFs exhibit considerable heterogeneity in size and location, complicating the selection of an optimal surgical strategy.

Although several endoscopic and open surgical techniques have been developed for TEF closure, outcomes remain suboptimal in complex cases. Endoscopic procedures are suitable for small, narrow fistulas, with reported success rates of 71.5% for recurrent TEF and 63.6% for acquired TEF.^15^, ^16^, ^17^ Open surgical approaches for complex cases may require specialized techniques, such as tissue grafting or reconstructive surgery, which require a good view of surgical field and well-designed strategies.^18^ Several surgical strategies were introduced in recent studies, including TEF division and repair, esophageal resection with reconstruction, repair with patch (muscle flap patch or thoracoacromial artery perforator flap patch), and esophageal diversion, but postoperative mortality (5.7%-18%), recurrent rate (4.5%-8.6%), and complication morbidity (42%-54.3%) cannot be ignored.^19^, ^20^, ^21^ Thus, a more effective salvage technique is urgently needed for complex TEF.

The slide tracheoplasty technique, first introduced in 1989 for congenital tracheal stenosis with a preferable outcome,^22^ has recently been advocated for the management of complex TEF in children.^23^ This technique provides excellent exposure of both the trachea and esophagus, enables complete resection of the fistulous tract, and facilitates robust reconstruction of both the trachea and esophagus. Herein, we present our institutional experience with slide tracheoplasty in 3 pediatric patients with complex TEF, with excellent outcomes reported at 1-year follow-up.

## Methods

The study was approved by the institutional review board of Children's Hospital of Fudan University (approval no. [2024]385; December 23, 2024). Individual patient consent was waived because of the retrospective nature of the study. Three patients who underwent slide tracheoplasty for TEF between January and April 2024 were included. This short inclusion period reflects the exceptional rarity of this condition and the fact that our center functions as a regional referral base for such complex cases. Medical records were reviewed to extract demographic data, TEF etiology, size and location, previous interventions, complications, and outcomes.

### Preoperative Management

**Figure 1:**
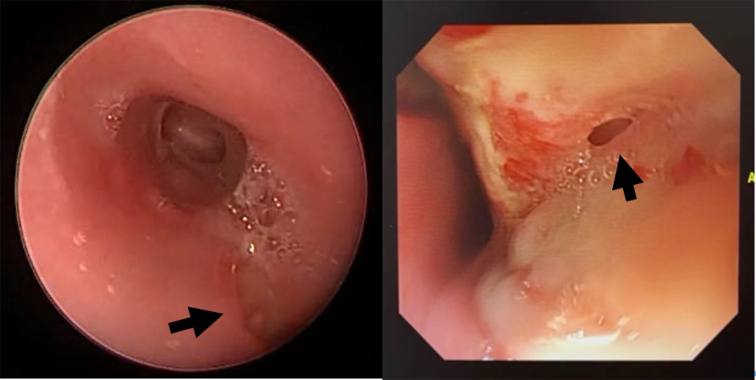
TEF was confirmed by MLB (*left*) and EGD (*right*) before surgery. *Black arrow*: TEF. *TEF*, Tracheoesophageal fistula; *MLB*, microlaryngoscopy and bronchoscopy; EGD, esophagogastroduodenoscopy.

**Figure 2:**
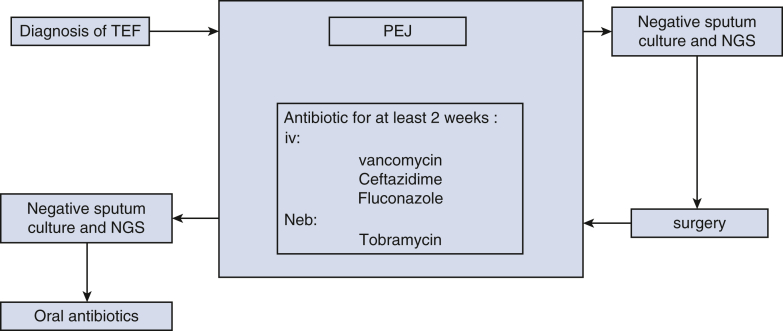
The perioperative anti-infective protocol. *TEF*, Tracheoesophageal fistula; *PEJ*, percutaneous endoscopy jejunostomy; *NGS*, next-generation sequencing.

All patients underwent microlaryngoscopy and bronchoscopy (MLB) and esophagogastroduodenoscopy (EGD) for definitive diagnosis (Figure 1). Percutaneous endoscopy jejunostomy was performed to avoid oral feeding and reduce infectious complications. A preoperative anti-infective regimen including intravenous vancomycin and meropenem, combined with nebulized tobramycin, was administered for at least 2 weeks, pending negative sputum culture results (Figure 2). Antibiotic therapy was adjusted according to sputum culture or next-generation sequencing results.

Airway management was carefully individualized. Spontaneous ventilation was maintained under total intravenous anesthesia with propofol-remifentanil during initial MLB. Topical lidocaine was applied to facilitate endotracheal intubation. The fistula was localized bronchoscopically, and the endotracheal tube was positioned distal to the fistula but above the carina under bronchoscopic guidance.

### Surgical Technique

**Figure 3:**
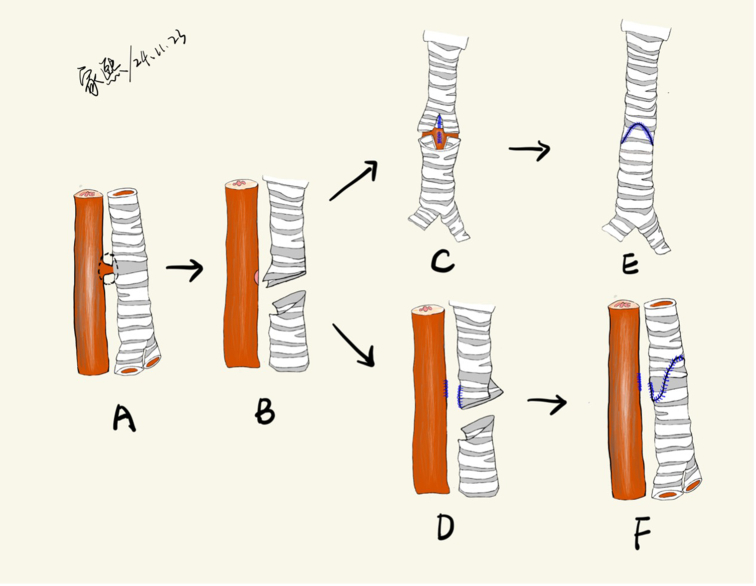
A, TEF in a sagittal plane view. B, In a sagittal plane view, the lesion of the TEF is removed. C, In a coronal plane view, the incisions of the esophagus and the posterior wall of the trachea are closed. The proximal tracheal end is split anteriorly approximately 1 cm, and the distal tracheal end is split posteriorly by an equal amount. D, In a sagittal plane view, the incisions of the esophagus and the posterior wall of the trachea are closed. The proximal tracheal end is split anteriorly approximately 1 cm, and the distal tracheal end is split posteriorly by an equal amount. E, In a coronal plane view, the trachea is closed. F, In a sagittal plane view, the trachea is closed. *TEF*, Tracheoesophageal fistula.

Patients were positioned supine with neck extension to optimize tracheal exposure (Video 1). Fistula condition was reconfirmed using intraoperative MLB and EGD. A median sternotomy was performed, and a pericardial patch was harvested for potential reinforcement. Cardiopulmonary bypass was established via cannulation of the ascending aorta, right atrial appendage, and inferior vena cava. This cannulation strategy was used to ensure a bloodless surgical field and facilitate optimal exposure of the posterior trachea. The trachea was carefully mobilized (Figure 3, *A*), with preservation of recurrent laryngeal nerves and lateral blood supply. Under bronchoscopic guidance, the fistula site was identified, and an oblique incision was made on the anterior tracheal wall. The fistulous tract was resected, and the esophageal defect was repaired primarily via direct closure (n = 1) or end-to-end anastomosis (n = 2) using continuous 3-0 or 4-0 VICRYL sutures (Ethicon) (Figure 3, *B*). A resulting V-shaped defect on the posterior tracheal wall was approximated with a continuous 5-0 or 6-0 polydioxanone suture (Ethicon) (Figure 3, *C* and *D*). Slide tracheoplasty was performed by creating a 1-cm incision in the proximal tracheal segment and a corresponding posterior incision in the distal segment. Anastomosis was completed using a continuous everting suture technique (Figure 3, *E* and *F*). No additional tissue flap (eg, pericardium, intercostal muscle) was interposed between the tracheal and esophageal suture lines. Interrupted 5-0 or 6-0 polydioxanone sutures with pericardial pledgets were placed every 5 mm to reduce anastomotic tension. An air leak test was performed underwater after lung inflation. Bronchoscopy was repeated to evaluate the anastomosis and rule out any luminal obstruction before reinstituting ventilation. Inversion of the tracheal mucosa was corrected with entire-layer suture or even resuture to minimize stenosis risk. It was confirmed that the endotracheal tube tip was positioned distal to the fistula to ensure adequate ventilation.

### Postoperative Management

**Figure 4:**
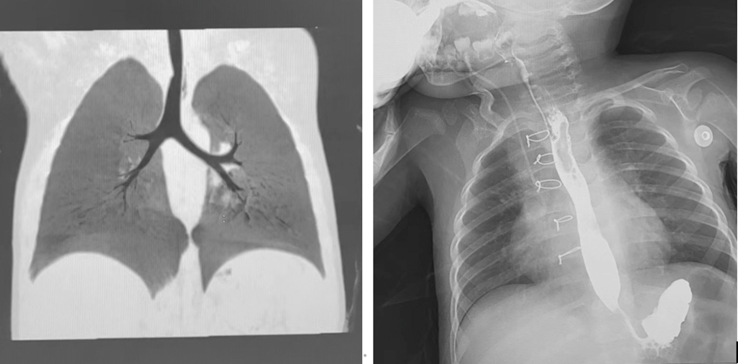
Follow-up via computed tomography (*left*) and gastrointestinal barium angiogram (*right*).

Routine MLB was performed on postoperative day 1 to access for bleeding or anastomotic leak. Extubation was achieved between postoperative days 1 to 3 based on clinical status. The post-operative anti-infective strategy is the same as the preoperative one (Figure 2). Percutaneous endoscopy jejunostomy was removed 1 month postoperatively after MLB confirms anastomotic integrity. Follow-up included computed tomography and barium esophagography to evaluate for complications or recurrence (Figure 4).

## Results

Results of the procedures are shown in Table 1. A description of cases follows herein.

**Table 1:** Summary of case characteristics

• Age • Gender • Weight	Etiology	Previous procedure	Location of TEF/diameter of TEF	CPB time, min	Mechanical ventilation time, h	CCU stay time, d	Complications	Follow-up
• 8 y • Male • 22.3 kg	• TEF after excision of bronchial cysts	• Endoscopic clipping × 2 • Bovine pericardial patch implantation	Distal one third of the trachea/5 mm	153	29	5	−	No recurrence No ES or TS No reintervention
• 10 mo • Female • 8.9 kg	• TEF after EA repair	• Endoscopic dilation of esophagus anastomosis • Endoscopic esophageal stent implantation and removal	Distal one third of the trachea/2 mm	204	67	5	−	No recurrence No ES or TS No reintervention
• 18 mo • Female • 10.1 kg	• Button battery ingestion for 4 d	• Removal of the battery with bronchoscopy	Middle one third of the trachea/8 mm	129	19	4	−	No recurrence No ES or TS No reintervention

*TEF*, Tracheoesophageal fistula; *CPB*, cardiopulmonary bypass; *CCU*, critical care unit; *ES*, esophageal stenosis; *TS* tracheal stenosis.

### Case 1

An 8-year-old boy weighing 22.3 kg was admitted to our hospital with complaints of cough, fever, and choking during liquid intake. He had undergone excision of a congenital bronchogenic cysts with bovine pericardial patch repair 4 months previously. A recurrent TEF was confirmed via MLB and EGD. The fistula was located in the distal one-third trachea and measured 5 mm in diameter. Two attempted endoscopic procedures were unsuccessful. After a 2-week anti-infective protocol, infection was controlled. Given the extensive scarring and the long segment of trachea requiring resection, slide tracheoplasty was performed primarily to achieve a tension-free anastomosis and ensure a robust repair, rather than to address luminal size. The procedure was performed on hospital day 38. Cardiopulmonary bypass (CPB) time was 153 minutes, and postoperative mechanical ventilation time was required for 29 hours. The patient remained in the cardiac care unit (CCU) for 5 days and was discharged after a total hospitalization of 54 days. At 9-month follow-up, there was no evidence of TEF recurrence or tracheal/esophageal stenosis.

### Case 2

A 10-month-old girl weighing 8.9 kg was admitted with cough, fever, and choking during liquid intake. She has been diagnosed with type III esophagus atresia and underwent primary repair at 2 weeks of age. Postoperative evaluation via MLB, EGD and barium esophagography revealed recurrent TEF and esophagus stenosis, accompanied by an esophageal diverticulum and tracheal membranous prolapse causing respiratory symptoms. The fistula was located in the distal one-third trachea and measured 2 mm. Endoscopic esophageal dilation and stenting failed to close the TEF. After infection control with an anti-infective protocol, slide tracheoplasty was performed on hospital day 23. CPB time was 204 minutes, and postoperative mechanical ventilation was maintained for 67 hours. CCU stay was 5 days, with total hospitalization lasting 45 days. At 6-month follow-up, the patient showed no recurrence or stenosis.

### Case 3

An 18-month-old girl weighing 10.1 kg presented with cough and fever 1 week after the ingestion of a button battery. The battery was removed via bronchoscopy 3 days before admission. TEF was confirmed using MLB and EGD, located in the middle one-third trachea, with a diameter of 8 mm. After infection control with an anti-infective protocol, slide tracheoplasty was performed on hospital day 17. CPB time was 129 minutes, with postoperative mechanical ventilation for 19 hours. The patient spent 4 days in the CCU and was discharged after 40 days. At 5-month follow-up, there was no recurrence of TEF or evidence of tracheal/esophageal narrowing.

## Discussion

Slide tracheoplasty has demonstrated high success rates in managing a broad spectrum of tracheal diseases, including congenital tracheal stenosis, complete tracheal rings, and tracheal sleeve malformations.^24^, ^25^, ^26^, ^27^, ^28^ Given its capacity to provide excellent exposure of both the trachea and the esophagus, this technique has been adapted for the repair of complex TEF. Kennedy and colleagues^23^ reported a series of 27 patients undergoing slide tracheoplasty for TEF, with a recurrence rate of 7.4%. Since January 2024, our institution has used slide tracheoplasty in the management of complex TEF. Our series does not seek to challenge their conclusions but rather to validate and extend them in several key areas. First, whereas their cohort encompassed a broad spectrum, our cases provide a focused look at its application in the most desperate salvage situations following multiple failed repairs. Second, we outline a standardized perioperative protocol that may contribute to preventing common complications such as granuloma formation. Thus, our work complements the existing literature by providing deep dives into specific, high-stakes applications of this versatile technique.

The decision to proceed with slide tracheoplasty warrants careful consideration, because it is an extensive procedure that may compromise future surgical options. In our cases, 2 patients had previously failed endoscopic and open thoracic repairs, whereas the third presented with a large, corrosive TEF secondary to button battery ingestion. Although a recurrent TEF after previous repair represents a classic scenario of complex tissue injury, the spectrum of “complexity” is broader. As demonstrated by our patient with a battery-induced fistula, severe acquired tissue necrosis from caustic ingestion creates a similarly formidable challenge. Slide tracheoplasty technique proves its versatility here, serving as a powerful single-stage solution not just for surgical failures but for any fistula where extensive tissue loss precludes conventional repair. This expands the potential indications for the technique to include other causes of destructive airway injury. Therefore, we suggest expanding indications for slide tracheoplasty with prudence to include (1) recurrent TEFs refractory to conventional thoracic or endoscpic approaches^23^ and (2) acquired TEFs from severe trauma or caustic ingestion (eg, battery). Avsar and colleagues^29^ successfully used slide technique in 4 cases of battery-induced TEFs and advocated for operative rather than conservative management in such scenarios.

Recurrence rates after repair of esophageal atresia with or without TEF range from 3% to 14%.^6^^,^^30^, ^31^, ^32^ Anastomosis leak is a leading cause of recurrent TEF,^33^^,^^34^ with reported incidence ranging from 7% to 26.5%.^35^, ^36^, ^37^ Among these, 45.8% may progress to recurrence.^32^ To mitigate this risk, we implemented several technical strategies during slide tracheoplasty. First, complete resection of the fistulous tract ensures removal of scarred or inflamed tissue, addressing the underlying pathology and reduce the risk of trachea stenosis, particularly in cases with membranous prolapse of the posterior tracheal wall. Second, an effective anti-infection protocol was utilized to control preoperative and postoperative infection of the TEF. Third, given that the esophageal and tracheal anastomosis lines are not at the same level, the breakdown of one closure line should not affect the other one. Last but not least, interrupted pericardial-pledget sutures were used to reinforce the tracheal anastomosis. It is worth noting that our technique did not use a tissue interposition graft, which is a common strategy to prevent recurrence. Our favorable outcomes without this step suggest that a secure, well-vascularized anastomosis may be sufficient in select cases, though the routine use of a pericardial flap remains a prudent option practiced by many centers.^18^

All procedures in this series were performed via sternotomy with CPB, which may raise concerns regarding CPB-related complications such as bleeding risk and cardiac injury, particularly when pericardium is harvested, potentially complicating future reoperations. Nevertheless, compared with a cervical approach, sternotomy approach provide superior exposure for mobilizing the trachea and the esophagus, facilitates tension-free anastomosis, and is especially advantageous for fistulas located in the distal one third of the trachea.^38^ Furthermore, it allows concurrent management of associated esophageal pathologies, such as diverticulum.

Although this series demonstrates the utility of slide tracheoplasty in complex fistulas, its role in the primary repair of neonates warrants further investigation. The technique is theoretically feasible and widely used for congenital tracheal stenosis in infants.^25^ However, for a simple type C tracheoesophageal fistula, the standard primary repair remains the procedure of choice due to its established efficacy and lower complexity. We propose that primary slide tracheoplasty may be best reserved for select cases involving ultra-long-gap atresia, associated congenital tracheal stenosis, or other factors that preclude a safe, tension-free conventional anastomosis.

Our study is limited by its small sample size and short inclusion period, which is inherent to the rarity of the condition and the highly specialized nature of the intervention. However, the consecutive nature of these referrals and the consistency of our outcomes provide a meaningful foundation for reporting this technique. The retrospective design introduces inherent limitations. Future multi-institutional studies with extended follow-up are needed to validate long-term outcomes.

## Conclusions

Slide tracheoplasty represents a successful and versatile technique for managing complex tracheoesophageal fistula. Further research is warranted to evaluate its long-term efficacy and broader applicability.

## Conflict of Interest Statement

The authors reported no conflicts of interest.

The *Journal* policy requires editors and reviewers to disclose conflicts of interest and to decline handling or reviewing manuscripts for which they may have a conflict of interest. The editors and reviewers of this article have no conflicts of interest.

## Supplementary Data

Video 1 After the trachea is incised, the lesion of the TEF is exposed and then removed; The incision of the esophagus is closed. The proximal tracheal end is split anteriorly approximately 1 cm, and the distal tracheal end is split posteriorly by an equal amount. The posterior wall and the anterior wall of the trachea are closed. Video available at: https://www.jtcvs.org/article/S2666-2507(25)00455-9/fulltext.
