# Supplementary figures and images for: Slide tracheoplasty for repair of complex tracheoesophageal fistulas in children: A salvage technique
Source: JTCVS Tech. 2025 Oct 13;34:195–202. doi: 10.1016/j.xjtc.2025.09.032 (PMC12683053; doi:10.1016/j.xjtc.2025.09.032)

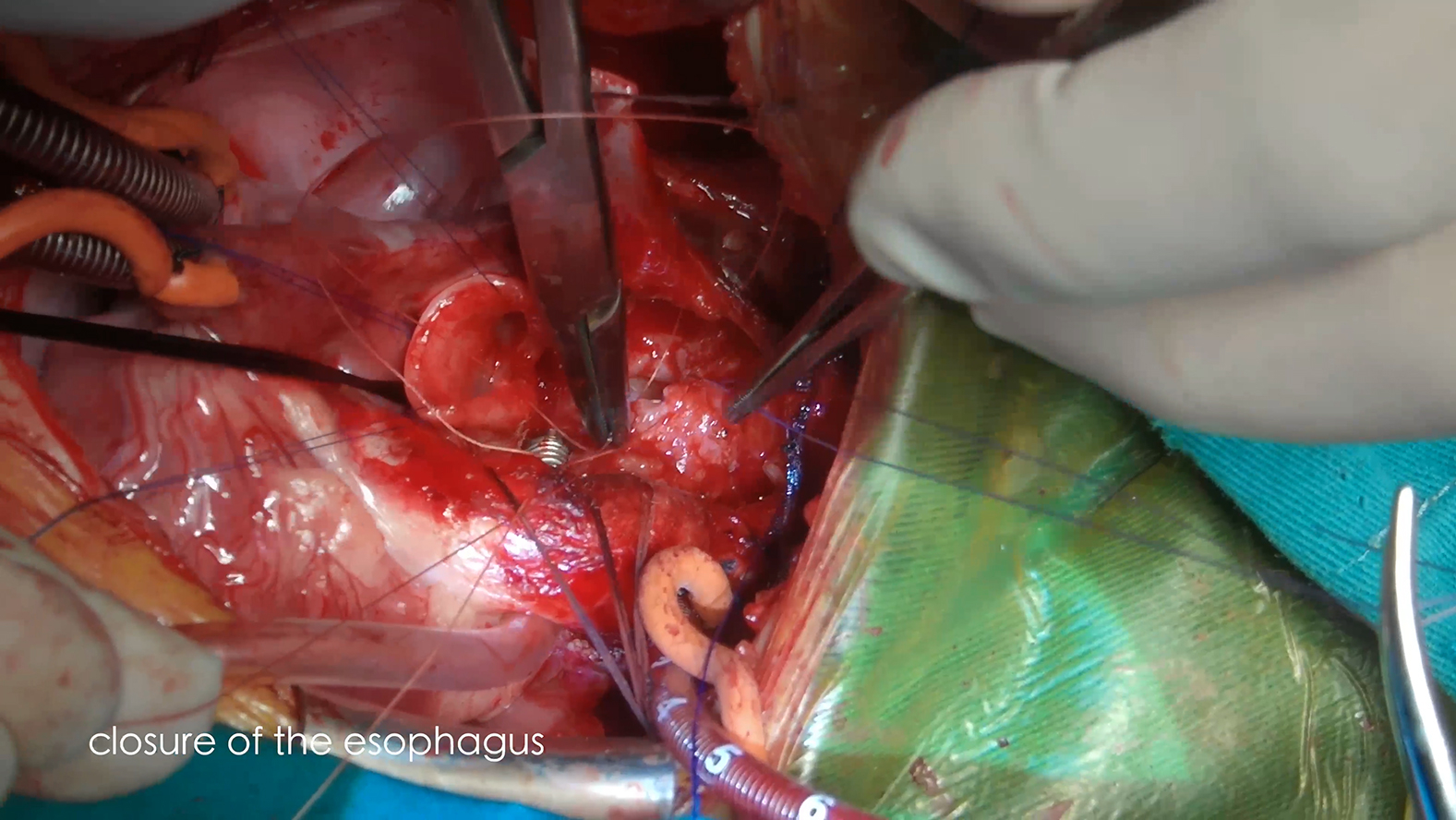

Supplement: Video 1 — After the trachea is incised, the lesion of the TEF is exposed and then removed; The incision of the esophagus is closed. The proximal tracheal end is split anteriorly approximately 1 cm, and the distal tracheal end is split posteriorly by an equal amount. The posterior wall and the anterior wall of the trachea are closed. Video available at: https://www.jtcvs.org/article/S2666-2507(25)00455-9/fulltext. [file fx2.jpg]
